# Self-Interest versus Group-Interest in Antiviral Control

**DOI:** 10.1371/journal.pone.0001558

**Published:** 2008-02-13

**Authors:** Michiel van Boven, Don Klinkenberg, Ido Pen, Franz J. Weissing, Hans Heesterbeek

**Affiliations:** 1 Faculty of Veterinary Medicine, Utrecht University, Utrecht, The Netherlands; 2 Animal Sciences Group, Wageningen University and Research Centre, Lelystad, The Netherlands; 3 Centre for Ecological and Evolutionary Studies, University of Groningen, Haren, The Netherlands; McMaster University, Canada

## Abstract

Antiviral agents have been hailed to hold considerable promise for the treatment and prevention of emerging viral diseases like H5N1 avian influenza and SARS. However, antiviral drugs are not completely harmless, and the conditions under which individuals are willing to participate in a large-scale antiviral drug treatment program are as yet unknown. We provide population dynamical and game theoretical analyses of large-scale prophylactic antiviral treatment programs. Throughout we compare the antiviral control strategy that is optimal from the public health perspective with the control strategy that would evolve if individuals make their own, rational decisions. To this end we investigate the conditions under which a large-scale antiviral control program can prevent an epidemic, and we analyze at what point in an unfolding epidemic the risk of infection starts to outweigh the cost of antiviral treatment. This enables investigation of how the optimal control strategy is moulded by the efficacy of antiviral drugs, the risk of mortality by antiviral prophylaxis, and the transmissibility of the pathogen. Our analyses show that there can be a strong incentive for an individual to take less antiviral drugs than is optimal from the public health perspective. In particular, when public health asks for early and aggressive control to prevent or curb an emerging pathogen, for the individual antiviral drug treatment is attractive only when the risk of infection has become non-negligible. It is even possible that from a public health perspective a situation in which everybody takes antiviral drugs is optimal, while the process of individual choice leads to a situation where nobody is willing to take antiviral drugs.

## Introduction

Recent outbreaks of SARS and H5N1 influenza have underlined the threat that viruses in the animal reservoir pose to the human population. Fortunately, neither SARS nor H5N1 influenza have become endemic in humans. Nevertheless, these and other events have stressed the importance of being prepared for emerging and reemerging infectious diseases.

For most infectious diseases vaccination is the preferred control measure. Indeed, vaccines generally have proven highly efficacious, providing strong and long-lasting immunity against infection, disease, and transmission. However, in case of a previously unknown infectious disease a vaccine may not be readily available. For such emerging infectious diseases the control options are limited. This is especially so for viral pathogens that cannot be treated by effective antimicrobial drugs. For these pathogens the control options are restricted to case isolation and contact tracing, promotion of changes in behavior, vaccination using vaccines with poor efficacy, and antiviral drugs. Although the efficacy of currently available antiviral drugs is also far from perfect and although antiviral drugs provide protection for a short amount of time only, an advantage of antiviral drugs over vaccines is their broad spectrum of action [1]–[5].

For a newly arising viral infectious disease it may be possible to contain an outbreak at an early stage by means of a large-scale antiviral control program if the control program is started early and has high compliance rates, if the efficacy of antiviral drugs is sufficiently high, and if the transmissibility of the pathogen is sufficiently low [6]–[8]. Hence, it seems logical that all efforts should be geared towards early control of an outbreak. However, whether people will cooperate with such a containment strategy is not known. Probably, the willingness to participate in a control program depends on the (perceived) risk of infection and the consequences of the subsequent disease as compared to the (perceived) risk of taking antiviral drugs. If there are adverse effects of taking antiviral drugs it may well be that people will only be inclined to start taking antiviral drugs once the risk of infection becomes non-negligible.

In this paper we employ population dynamical and game theoretical analyses to investigate (i) under which conditions an antiviral control program can prevent an epidemic, and (ii) when people should consider taking antiviral drugs. With regard to the latter question we take two perspectives. First, we focus on the public health officer whose goal it is to minimize the total amount of damage caused by both infection and prophylactic antiviral treatment. In a second step we then compare the strategy that is optimal from the point of view of the population as a whole with the strategy that would evolve if individuals pursue their own interest.

The dilemma that an individual faces is the following. Should you take your chances and refuse antiviral prophylaxis? The price that you may have to pay upon infection may be high (death in its most extreme consequence). The potential reward is that once you have successfully recovered from infection you also reap the benefit of long-lasting immunity. The alternative is that you take antiviral drugs and so avoid the potentially high cost of infection. The drawback of this option is that you may have to take antivirals for a prolonged period of time. This has negative side-effects in the short term [9], and may in the long run also not be harmless.

The situation is complicated by the fact that an individual's risk of infection does not only depend on whether or not the individual itself decides to take antiviral drugs but also on the decision of others. In the context of vaccination it is well known that in such a situation there can be a trend of decreasing willingness to participate in a control program, which will lead to strategies that are not optimal from the population perspective [10]–[14]. Here we ask whether similar phenomena occur in case of antiviral prophylaxis. While vaccination usually provides long-lasting and strong immunity after one or a few vaccination bouts, antiviral prophylactic therapy relies on the continuous administration of drugs. This implies that, in contrast with vaccination, individuals have more opportunities to adjust their actions to the situation in which they face themselves. Further, while the earlier studies focus on the relative perceived risk of infection as compared to vaccination, we consider infections and antiviral drugs that induce a real, albeit possibly small, risk of death. Throughout, our aim is to decipher how the optimal antiviral prophylactic control strategy is moulded by the transmissibility of the pathogen, by the risk associated with antiviral prophylaxis, and by the efficacy of antiviral drugs in reducing susceptibility, infectiousness, and mortality.

We would like to stress from the onset that we strive more for conceptual clarification than for the most precise representation of a specific system. In particular, all our analyses are based on the simplifying assumptions that individuals act rationally, have perfect information and foresight while they do not engage in projecting the epidemic, and that the details of population structure play a minor role. We are aware that these simplifying assumptions cannot be neglected in the real world, and we therefore do not believe that our model is suited to make quantitative predictions for any specific emerging infectious disease. Rather, our models serve a purpose by laying out, in an idealized context, the key factors shaping the interests of individuals and public health officers. In case of influenza vaccination others have investigated models with added layers of complexity with the goal to make quantitative predictions [13].

## Methods

**Figure 1:**
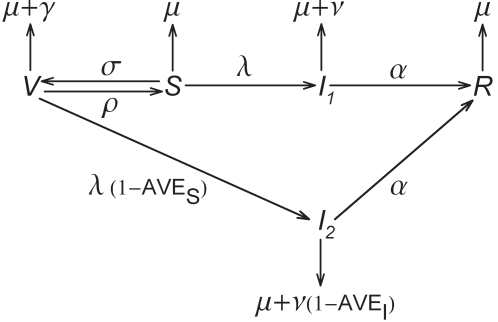
Schematic of the model. See text for details.

Stochastic and deterministic *SIRV*-type epidemic models in which individuals are susceptible (*S*), infected and infectious (*I*_1_ or *I*_2_), recovered and immune (*R*), or (partially) protected against infection by antiviral prophylactic treatment (*V*) form the basis of the analyses. Figure 1 shows a schematic of the model. Details are given in Text S1. Throughout susceptible individuals enter the population by birth. The (natural) death rate of susceptible and recovered individuals is denoted by *μ*, and the excess mortality while on antiviral treatment is given by *γ*. Hence, life expectancy is *μ* ^−1^ in the absence of infection and antiviral control, and (*μ*+*γ*)^−1^ while on antiviral drugs. In the infected classes (*I*_1_ and *I*_2_) the excess death rates resulting from infection are given by *ν* and *ν*(1−AVE*_I_*), where AVE*_I_* is the antiviral efficacy for infectiousness and virulence. In the following, *γ* and *ν* will be referred to succinctly as the cost of antiviral prophylaxis and infection. From the susceptible class individuals move to the protected and infected classes at rates *σ* and *λ*. The parameter *λ* is colloquially called the force of infection, and it depends on the prevalence of infection (Text S1). Individuals in class *V* are infected at a reduced rate *λ*(1−AVE*_S_*), where AVE*_S_* denotes the antiviral efficacy for susceptibility. This implies that individuals in class *V* cannot be infected at all if AVE*_S_* = 1, while the antiviral drug provides no protection against infection if AVE*_S_* = 0. Finally, the rates of recovery and non-compliance are given by *α* and *ρ*, respectively. An overview of the model parameters and their default values is given in Table 1. Details of the model analyses are provided in Text S1.

**Table 1:** Model parameters and default parameter values.

Parameter	Default value	Description
μ	0.0125 (yr^−1^)	natural death rate
β	150 (yr^−1^)	infection rate parameter
α	50 (yr^−1^)	recovery rate
ν	1 (yr^−1^)	infection induced mortality rate
γ	0.0001 (yr^−1^)	antiviral prophylaxis induced mortality rate
σ	variable	rate of enrollment on antiviral prophylactic drugs
ρ	1 (yr^−1^)	rate of non-compliance
*AVE_S_*	1 or 0.3	antiviral efficacy for susceptibility
*AVE_I_*	0.8	antiviral efficacy for virulence and infectiousness
*R*_0_	2.94	basic reproduction number (σ = 0)
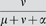	≈0.02	probability of death by infection without antiviral treatment
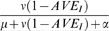	≈0.004	probability of death by infection while on antiviral treatment
*_e_* ^−*μ*^_(1−*e*_^−*γ*^_)_	≈0.0001	per year probability of antiviral treatment induced death

## Results

### Prevention

When will a prophylactic antiviral control program be able to prevent an epidemic? Several studies have addressed this question using simulations of metapopulation models that include household structure and other population and pathogen details [6]–[8]. Here we focus on a basic model for a large well-mixed population. To evaluate whether successful invasion of the pathogen is possible we calculate the (basic) reproduction number (denoted by *R*_0_), which gives the number of new infections caused by a single infected individual in a population of uninfected individuals in the early stages of an outbreak [15]. If *R*_0_>1 the pathogen can invade a population in which it is not yet present, while it cannot if *R*_0_<1. In the case that the pathogen-induced mortality is such that it hardly affects the infectious period, the reproduction number is given by
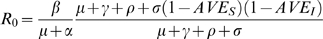
(1)(see Text S1 for a derivation). The first factor in equation (1) gives the reproduction number in a population consisting of susceptible individuals only 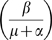, while the second factor represents the sum of the fractions of individuals in the susceptible and protected classes 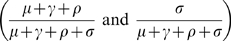, where the individuals in the protected class are weighed by their relative susceptibility (1−AVE*_S_*) and relative infectiousness if infected (1−AVE*_I_*). Notice that the reproduction number increases with increasing pathogen transmissibility (*β*) and length of the infectious period ((*μ*+*α*)^−1^), and with increasing rate at which individuals leave the protected class (*μ*+*γ*+*ρ*). The reproduction number decreases with increasing rate of antiviral prophylaxis (*σ*).

Equation (1) shows that the pathogen cannot invade the population if the rate of antiviral prophylaxis exceeds a certain critical rate of antiviral prophylactic therapy σ*_c_*, which is given by the solution of the equation *R*_0_ = 1. For the default parameter values (Table 1) it turns out that the critical rate of antiviral prophylaxis is *σ_c_* = 1.96 (*yr* ^−1^) if antiviral drugs provide complete protection against infection (AVE*_S_* = 1). This implies that approximately two-thirds of the population needs to be protected against infection by antiviral prophylaxis in order to prevent an epidemic. This fraction increases if antiviral drugs provide partial protection against infection and subsequent transmission.

### Early control

**Figure 2:**
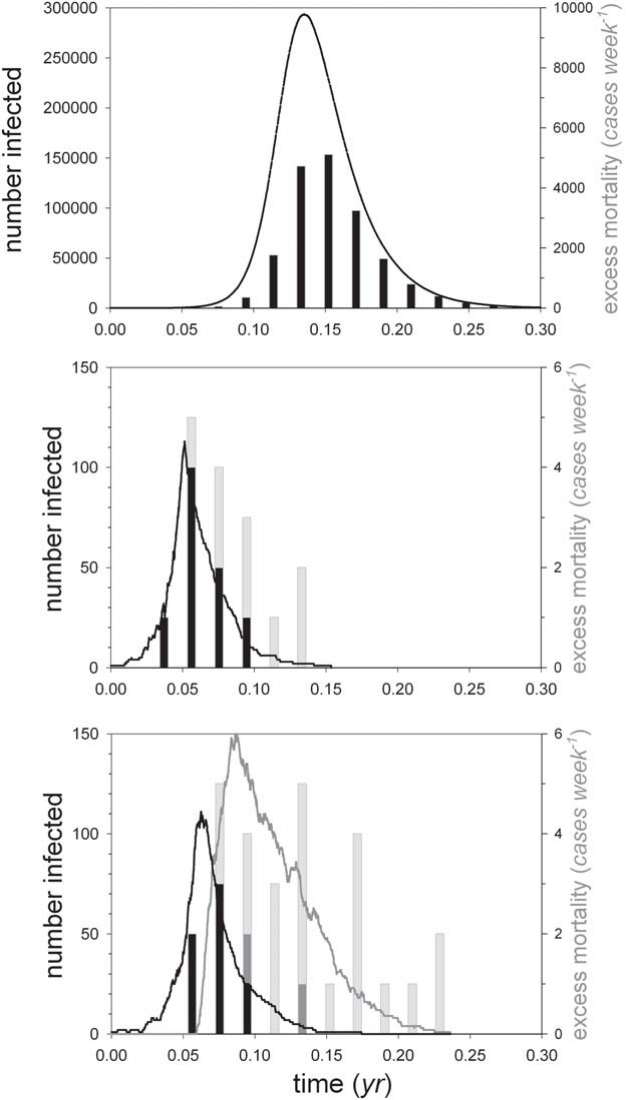
Simulations of an epidemic in a population of 10^6^ individuals. The top panel shows the number of infected individuals (line) and mortality (bars) in the absence of an antiviral control program. The middle and bottom panel show simulations in case of a control program with an antiviral drug that provides complete (*AVE_S_* = 1) and imperfect (*AVE_S_* = 0.3, *AVE_I_* = 0.8) protection, respectively. Black bars refer to deaths caused by type 1 infection, grey bars to deaths caused by type 2 infections, and light bars to deaths caused by antiviral prophylaxis. The grey line in the bottom panel shows the number of infected individuals who were taking antiviral prophylaxis.

Is it possible to prevent a major epidemic with an antiviral response that is started quickly after an introduction of the pathogen? To answer this question we performed stochastic simulations in which an antiviral response is initiated after a certain number of individuals are infected (see Text S1 for details). Figure 2 shows three representative simulation runs of an epidemic in a large but finite population (10^6^ individuals). If no control measures are put into place (top panel), epidemiological theory informs that a large epidemic will unfold with probability 
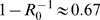
, and the fraction of individuals that is infected once the epidemic has taken off is roughly given by the solution of the final size equation (log(1−*x*) = −*R*_0_ *x*) [15]. For the default parameter values this means that 94% of the population will be infected, of which some 2% will die from the sequelae of infection. In a population of one million individuals this implies that more than 18,000 individuals will die during the course of an epidemic.

The situation is completely different if a large-scale antiviral prophylactic control program is initiated once a certain number of individuals is infected. The middle and bottom panels of Figure 2 show simulations in case of a perfect and imperfect antiviral drug, respectively. In both panels we assume that antiviral control is started once 100 individuals are infected, and that no individuals are exempted from antiviral drug treatment. The middle panel shows that even though the cost of antiviral prophylaxis is much smaller than the cost of infection (Table 1) the number of individuals that has died by antiviral treatment at the end of the epidemic equals the number that has died from infection (8 individuals). If the antiviral drug is imperfect (bottom panel), the duration of the epidemic is considerably longer, and many more individuals will have died from antiviral treatment than from infection (22 versus 6). Still, the total number of deaths is orders of magnitude smaller than in the case that no antiviral control program is effective.

The simulations of Figure 2 illustrate two general phenomena. First, the number of infections and deaths is reduced dramatically by an antiviral control program that is able to successfully contain an epidemic [6]–[8]. Second, while the number of deaths caused by infection is proportional to the number of infected individuals (which is relatively small at an early stage of an epidemic), the number of deaths related to antiviral prophylaxis is proportional to the number of individuals that have taken antiviral drugs. The latter may be quite large, and is probably on the order of total population size. Hence, even though the individual risk of antiviral prophylaxis is small and large-scale antiviral prophylactic control appears to be the rational strategy, it may well be that the number of deaths induced by antiviral treatment exceeds the number of deaths induced by infection. Motivated by these observations we investigate in the following (i) the conditions under which a large-scale antiviral control program is able to halt an epidemic, and (ii) the conditions under which rational individuals are willing to take antiviral drugs.

### The critical force of infection

At what point in an unfolding epidemic does the risk of infection exceed the risk of antiviral treatment? This question is relevant because it determines the incentive for individuals to take antiviral drugs. We focus on the probability that an individual is alive after a certain time (the horizon) given that it is initially susceptible or (partially) protected against infection by antiviral control. If the probability to remain alive is larger when initially susceptible than when under antiviral treatment, the best option is not to take antiviral drugs. Otherwise the reverse is true. The formal analyses are given in Text S1. Here we summarize the main findings.

**Figure 3:**
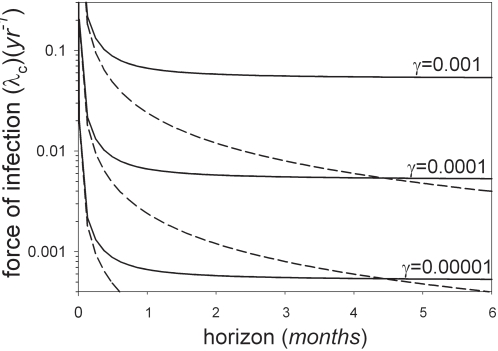
The critical force of infection at which the risk of infection equals the risk of antiviral prophylaxis as a function of the horizon. The antiviral death rate is varied from *γ* = 0.00001 (*yr* ^−1^) to γ = 0.001 (*yr* ^−1^). The dotted lines show the approximation based on equation (A7) of the Supporting Information.

Figure 3 shows the relation between the horizon and the critical force of infection which determines whether individuals should or should not take antiviral drugs. The critical force of infection strongly depends on the cost of antiviral treatment. In fact, if the risk of antiviral treatment is low (*γ* = 0.00001 (*yr* ^−1^)), the critical force of infection ranges from 0.0005 (*yr* ^−1^) to 0.0007 (*yr* ^−1^) if the horizon is one month or longer. Assuming a standard relation between the the force of infection and the prevalence of infection (see Text S1) this implies that individuals should consider taking antiviral drugs once the prevalence of infection is in the range 3.4*10^−6^ to 4.1*10^−6^. Hence, taking antiviral drugs is the rational strategy once three to four persons are infected in a population of one million. If the cost of antiviral treatment is increased one order of magnitude the critical force of infection is also increased by approximately one order of magnitude, and the risk of infection outweighs the risk of antiviral prophylaxis if approximately 40 or more individuals are infected. If the cost of antiviral treatment increases another order of magnitude (*γ* = 0.001 (*yr* ^−1^)) the critical force of infection again increases by an order of magnitude, and taking antiviral drugs is the rational strategy if 400 or more individuals are infected.

**Figure 4:**
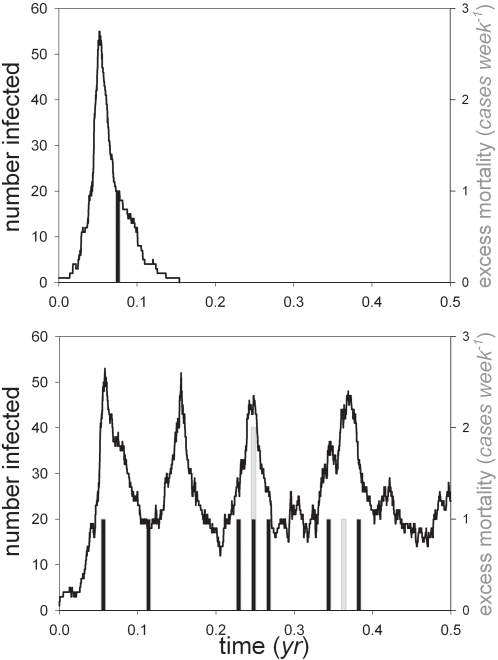
Simulations of an epidemic in a population of 10^6^ individuals when the decision whether or not to take antiviral drugs is determined by the critical force of infection (Figure 3). The top and bottom panel show representative simulation runs in case of low (*ρ* = 1 (*yr* ^−1^)) and high (*ρ* = 6 (*yr* ^−1^)) rates of non-compliance, respectively. Other parameters are as in Table 1.

Figure 4 shows two representative simulation runs of the pathogen dynamics if all individuals base their decision whether or not to take antiviral drugs on the critical force of infection. If the force of infection remains smaller than the critical force of infection (i.e. if the number of infected individuals remains below a certain threshold) then there is no incentive to take antiviral drugs, while the reverse is true if the force of infection exceeds the critical force of infection. For the default parameter values the threshold is reached when 45 individuals are infected in a population of a million. The top panel shows that an unfolding epidemic in principle can be halted with little cost of human lives, while the bottom panel illustrates that high compliance rates are essential for successful early control, preventing a rapid buildup of susceptible individuals after the number of infected individuals has dropped below the threshold.

### Late control

Now let us suppose that attempts to control an outbreak at an early stage have been unsuccessful. In this case it is still of interest to determine whether and under which conditions antiviral prophylaxis should be part of a strategy aimed at pathogen eradication or containment. If antiviral treatment provides complete protection against infection (AVE*_S_* = 1) the equilibrium prevalence of infection decreases monotonically with increasing rate of antiviral control, up to the point where the pathogen cannot persist (Text S1). Furthermore, as long as the risk of antiviral prophylaxis remains small its precise value hardly affects the prevalence of infection. This is due to the fact that mortality related to antiviral treatment is negligible in comparison with natural mortality.

Although the risk of antiviral prophylaxis may have a negligible effect on the prevalence of infection, it does affect excess mortality at equilibrium induced by infection and antiviral treatment. In fact, in case of a perfect antiviral drug excess mortality decreases with increasing rate of antiviral prophylaxis if
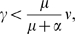
(2)i.e. if the cost of antiviral prophylaxis is small compared to the cost of infection. This implies that at equilibrium excess mortality is lowest at the point where the pathogen is just driven to extinction. If, on the other hand, inequality (2) is reversed, excess mortality increases with increasing rate of antiviral prophylaxis, so that excess mortality is lowest if no antiviral drugs are taken at all. For the default parameter values the right-hand side of inequality (2) equals 0.00025 (*yr* ^−1^), while the cost of antiviral prophylaxis is *γ* = 0.0001 (*yr* ^−1^). Hence, in our default scenario excess mortality decreases with increasing rate of antiviral control up to the point where the pathogen is just unable to persist (*σ* = 1.96 (*yr* ^−1^)).

### The individual versus population perspective

Next we turn our attention to the different perspectives of the individual versus the public health officer. Our focus is on the antiviral treatment rate that minimizes excess mortality. Minimizing this quantity with respect to the antiviral treatment rate yields the strategy that is optimal from the population perspective. Throughout this section and the next we assume that the pathogen is endemically present at the population dynamical equilibrium. As we have argued above, the optimal population strategy is such that the pathogen is just unable to persist if the risk of antiviral prophylaxis is small (i.e. if (2) is satisfied). Otherwise, the optimal population strategy is not to take antiviral drugs at all (Text S1)

In Text S1 we also show how excess mortality of a small group of individuals with antiviral treatment rate *σ_y_* is calculated in a population where the majority of individuals take antiviral drugs at a rate *σ_x_*. This allows one to determine the antiviral treatment strategy that will evolve at the population level by the process of individual choice. The optimal population and individual rates of antiviral treatment at the population level will be denoted by 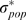 and 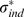, respectively.

**Figure 5:**
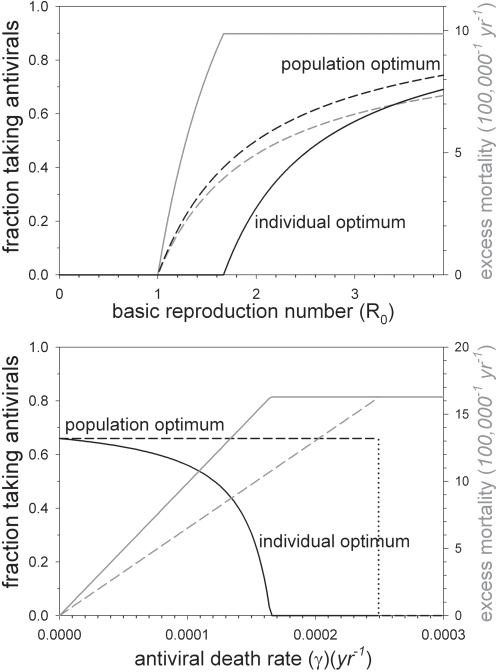
The optimal amount of antiviral prophylaxis at equilibrium as a function of the basic reproduction number (top panel) and the antiviral death rate (bottom panel) in case of a perfect antiviral drug (*AVE_S_* = 1). The top panel shows the optimal antiviral control rate from the population and individual perspective (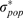: black dashed line; 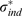: black solid line). The grey lines give the associated excess mortality. The bottom panel shows the same quantities as a function of the antiviral death rate (*γ*).

Figure 5 shows the results of a systematic investigation of the relation between the model parameters and the fractions of individuals taking antiviral drugs (which are determined by the antiviral control rates 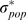 or 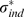). The top panel shows the fraction of individuals taking antiviral drugs (black lines) and the associated excess mortality (grey lines) as a function of pathogen transmissibility. If transmissibility is low (*β*<51 (*yr* ^−1^)) the pathogen cannot persist, and there is no need to take antiviral drugs. If transmissibility is intermediate there is a positive population optimum (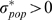) which ensures eradication of the pathogen, while the individual optimum is still zero (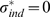). If transmissibility is high both the population and individual control rates are positive, although eradication is only achieved by the optimal population control rate.

The bottom panel of Figure 5 illustrates how the fractions of individuals taking antiviral drugs depend on the antiviral death rate. Not surprisingly, if antiviral drugs incur no cost (*γ* = 0) then both the population and individual optimal control rates are such that the pathogen is driven to extinction. For the default parameter values this is achieved if at least two-third of the population is on antiviral drug treatment (*σ*≥1.96). If there is a cost to antiviral treatment, then the best option is to drive the pathogen to extinction if one takes the population perspective, until the risk of antiviral prophylaxis exceeds the risk of infection at the endemic equilibrium with no antiviral control (*γ*>0.00025 (*yr* ^−1^)). Alternatively, if all individuals are allowed to flexibly adjust their own strategy, the optimal rate of antiviral prophylaxis decreases gradually with increasing antiviral death rate. In this case the optimal rate of antiviral prophylaxis is zero if γ>0.00016 (*yr* ^−1^). Notice that for intermediate cost of antiviral treatment the public health officer favours a strategy that is aimed at eradicating the disease, while the process of individual choice leads to a situation where nobody is willing to take antiviral drugs.

### Imperfect antiviral drugs

Unfortunately, to date there are no antiviral drugs that provide complete protection against infection and disease. For instance, an analysis of two recent trials with the antiviral drug oseltamivir shows that it provides little protection against infection with influenza, and moderate protection against subsequent shedding and disease [16]. Therefore, we will in this section study the consequences of antiviral prophylactic treatment with an imperfect antiviral drug. In the analyses below we take *AVE_S_* = 0.3 and *AVE_I_* = 0.8 as default parameter values [16].

**Figure 6:**
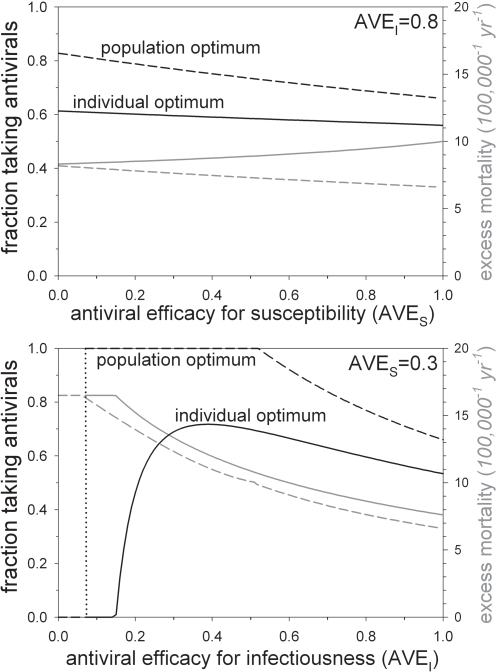
The optimal amount of imperfect antiviral prophylactic treatment at equilibrium as a function of the antiviral efficacy for susceptibility and infectiousness. The top panel shows the optimal antiviral control rate from the perspective of the individual and the public health officer (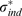: black solid line; 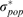: black dashed line) as a function of the antiviral efficacy for susceptibility. The grey lines give the associated excess mortality. The bottom panel shows the same quantities as a function of the antiviral efficacy for infectiousness. Parameter values are as in Table 1 with *AVE_I_* = 0.8 (top panel) and *AVE_S_* = 0.3 (bottom panel).

The relation between the antiviral efficacies for susceptibility and infectiousness, and the optimal rates of antiviral control is investigated in Figure 6. The top panel shows the relation between the antiviral efficacy for susceptibility and the optimal fraction of individuals taking antiviral drugs, assuming that antiviral efficacy for infectiousness and virulence is fixed at *AVE_I_* = 0.8. The fact that the optimal fractions taking antiviral drugs decrease with increasing antiviral efficacy for susceptibility can be understood as follows. A decrease in the antiviral efficacy for susceptibility renders the antiviral drug less effective. However, since the cost of antiviral treatment is low while the antiviral efficacy for infectiousness is relatively high the rational strategy is to eradicate the pathogen if one takes the population perspective. With decreasing antiviral efficacy for susceptibility this is achieved by increasing the rate of antiviral control. Interestingly, the top panel indicates that if one takes the individual perspective excess mortality is highest if antiviral drugs provide complete protection against infection, since then the optimal control rate is lowest.

The bottom panel of Figure 6 shows the relation between the optimal fractions of individuals taking antiviral drugs as a function of the antiviral efficacy for infectiousness. The antiviral efficacy for susceptibility is fixed at *AVE_S_* = 0.3. The picture in this panel is more complicated than in the top panel. In particular, eradication of the pathogen is not feasible if the antiviral efficacy for infectiousness drops below a critical value (*AVE_I_*<0.51). If the antiviral efficacy for infectiousness is just above this critical value it still is the best strategy to drive the pathogen to extinction if one takes the population perspective. However, this can only be achieved if almost all individuals are in the protected class (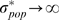). If, on the other hand, the antiviral efficacy for infectiousness is low (*AVE_I_*<0.07) it is better not to take antiviral drugs at all (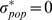) as the benefit of taking antiviral drugs do not outweigh the cost. In this region of parameter space both optimal control rates are zero. In the intermediate parameter regime (0.07<*AVE_I_*<0.51) it is not possible to achieve eradication, but it may nevertheless be wise to take antiviral drugs. In fact, taking the population perspective, it is always better to be (partially) protected by antiviral drugs than to be fully susceptible in this region of parameter space (i.e. 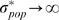), even though eradication is not possible.

## Discussion

Intuitively, it may seem that one should consider antiviral treatment when faced with a highly transmissible pathogen that can kill its host. However, this line of reasoning may be inaccurate. In particular, the notion that antiviral treatment is attractive because the drugs are relatively harmless and because a potentially large number of infection-induced deaths can be prevented is not necessarily true. Our analyses show that over the course of an epidemic the death toll can be considerable if no antiviral drugs are taken, and that the number of deaths is orders of magnitudes smaller if a large-scale antiviral control program is effective (Figure 2). However, the analyses also show that in an epidemic that is effectively controlled by a large-scale antiviral treatment program the majority of deaths result from the use of antiviral drugs. The intuitive explanation is that although the hazard of mortality by antiviral prophylaxis is small on the short-term individual level, the total death toll may be quite high as the number of individuals that must receive antiviral drugs for an effective control effort is probably on the order of total population size. Moreover, adding to this is the fact that in the face of an imminent threat it may prove necessary to continue taking antiviral drugs for a prolonged period.

In the early stages of an unfolding epidemic the probability of infection is still small. Consequently, individuals may be tempted to put off antiviral drug treatment until the prevalence of infection and hence the probability of infection has become non-negligible. Our analyses have shown that the critical force of infection that determines at what point individuals should start taking antiviral drugs depends strongly on the adverse effects of antiviral drug use (Figure 3). Hence, for purposes of successful prevention or early containment it is important that the adverse effects of antiviral drugs remain small.

Our analyses have revealed that, taking the population perspective, the best option is either to provide antiviral drugs up to the point where the pathogen is unable to invade and persist, or not to provide antiviral drugs at all (Figures 5–6), depending on the cost of antiviral treatment and the effectiveness of antiviral treatment. If, on the other hand, one takes the individual perspective there is an incentive to take less antiviral drugs than is optimal from the population perspective, and complete prevention or eradication of the disease is rarely possible.

Interestingly, the conflict between the individual and the public health officer appears to be most pronounced when the cost of antiviral drug treatment or the effectiveness of antiviral drugs in reducing the adverse effects of infection are intermediate (Figures 5–6). In fact, if the cost of antiviral prophylaxis is intermediate it is possible that public health favours an aggressive containment strategy that aims at pathogen eradication, while the process of individual choice leads to a situation in which nobody is willing to take antiviral drugs. On the other hand, if the cost of antiviral treatment is high or if the effectiveness of antiviral drugs in preventing the adverse effects of infection is very low, then the conflict may become small or disappear at all (Figures 5–6).

One aspect of our model that deserves special attention is that we have throughout assumed that the cost of both infection and antiviral treatment are in terms of an increased risk of death. This is convenient because it enables a straightforward comparison of the positive and negative consequences of infection and antiviral prophylaxis. However, although there is no question that human infections with SARS and H5N1 avian influenza bring along a risk of death, it is as yet unclear how severe the adverse effects of antiviral drug treatment may be. This is especially so for rare but possibly severe adverse effects. For instance, it is well-documented that oseltamivir (the neuraminidase inhibitor currently used to treat and prevent influenza infections) frequently leads to nausea and a number of less frequent adverse effects such as hepatitis and skin reactions [9], [17]. Recently, there have been suggestions of more serious adverse effects, including neuropsychiatric syndromes that may have contributed to a number of suicide events in Japan [18]–[19].

While we have used mortality as the currency to compare the costs and benefits of antiviral drug use, previous game theoretical studies of vaccination focused on the relative perceived risk of vaccination as compared to infection, and thereby also introduced a common currency to compare the costs and benefits of vaccination [10]–[14], [20]. Using relative perceived risk of vaccination as the basis of comparison has the advantage that it can be easily modeled. However, this approach also has some disadvantages as it assumes that the payoff loss for individuals who choose to vaccinate is a fixed quantity (the relative perceived cost of vaccination) which is unrelated to the actual number of adverse events in the population, while the payoff loss for individuals who choose not to vaccinate is proportional to the prevalence of infection, and so does not take into account the discounting of different costs and benefits (e.g., individuals who successfully recover from infection reap the long-term benefit of prolonged immunity). Ultimately, we believe that game theoretical models such as the one we have analyzed here should be refined to include the dynamics of human risk perception. In such models the perceived risks of infection and antiviral treatment are not static (as in [10]–[14]) but dynamically adjusted, being functions of the different types of adverse events (different types of morbidity, deaths) that actually occur in the population. Of course, how such dynamical human risk perceptions can or should be modeled is not straightforward, and would necessitate adding a fair bit of sociology to our epidemic-game theoretical model.

Throughout this paper we have made the simplifying assumptions that individuals and public health officer's act rationally, have perfect information and foresight, and that the details of population structure and antiviral drug action play a minor role. These assumptions were made in order to be able to keep the analyses manageable, and to be able focus in detail on the conflict of interest. We are, of course, aware that in the real world a variety of complicating factors play a role. Therefore, our study is not intended nor suited to make quantitative predictions, but it serves to explore how the public and individual interests are shaped by pathogen transmissibility, cost of antiviral treatment, and antiviral efficacy for susceptibility and infectiousness.

It would be interesting to extend the model in a number of directions in order to be able to make specific predictions for specific viral threats. For this purpose several steps should be taken. First, depending on the precise research question some form of population structure would probably need to be taken into account. For instance, if the goal were to decide how a limited supply of antiviral drugs is best distributed across different risk groups, the model would need to include different risk groups and take into account that the stockpile of antiviral drugs or vaccines is not infinitely large [21]-[22]. Alternatively, if the goal were to investigate whether local containment is possible by means of a targeted antiviral drug treatment program, it would be necessary to include spatial structure, household structure, and possible also workplace structure [6]–[8]. Overall, however, we believe that the present state of knowledge just barely suffices to make realistic quantitative predictions as to how effective a large-scale prophylactic antiviral drug program will be, let alone that it will be possible to make quantitative predictions when the dynamics of human choice are taken into account. This, of course, is not tantamount to saying that individual choice is unimportant.

Fortunately, none of the recent viral threats from the animal reservoir (avian influenza, SARS) has succeeded in getting a definitive foothold in the human population. As a consequence, the key epidemiological characteristics of the next emerging virus (transmissibility, infectious period, virulence) remain unknown. This is also largely true for rare but serious side-effects of antiviral drugs. This has rendered attempts to provide realistic predictions of the effectiveness of control measures such as antiviral treatment somewhat speculative [6]–[8]. Our model lacks much of the sophistication of the earlier models, and is not suited to make quantitative predictions. Rather, the analyses have laid out the principles guiding the decisions of rational individuals and public health officers when faced with an emerging viral threat for which antiviral drugs can be deployed as a first line of defense.

## Supporting Information

Text S1 Model structure and details of the model analyses
(0.10 MB PDF)
