# Supplementary material for: Self-Interest versus Group-Interest in Antiviral Control
Source: PLoS One. 2008 Feb 13;3(2):e1558. doi: 10.1371/journal.pone.0001558 (PMC2592701; doi:10.1371/journal.pone.0001558)
Supplement: Text S1 — Model structure and details of the model analyses (0.10 MB PDF) [file pone.0001558.s001.pdf]

Text S1. Self-interest versus group-interest in antiviral control

Michiel van Boven[1,2,*], Don Klinkenberg[1], Ido Pen[3], Franz J. Weissing[3] & Hans Heesterbeek[1]

Model structure

The analyses are based on a $SIRV$ type epidemic model in which individuals are classified as susceptible (S), infected and infectious (I_1 and I_2), recovered and permanently immune (R), and (partially) protected by antiviral treatment (V). The dynamics of the basic model is determined by ordinary differential equations for the numbers in the various classes. To streamline the analyses we assume initially that (i) births and deaths are not necessarily balanced, and (ii) the force of infection is constant. These two feedback loops are included at a later stage.

The dynamics of the model is governed by the following set of differential equations:

$$
\begin{aligned}
\frac{dS}{dt} &= b\,(S + I_1 + R + V + I_2) - \mu S + \rho V - (\lambda + \sigma)\,S \\
\frac{dI_1}{dt} &= -\,(\mu + \nu)\,I_1 + \lambda S - (\nu + \alpha)\,I_1 \\
\frac{dR}{dt} &= -\mu R + \alpha\,(I_1 + I_2) \\
\frac{dV}{dt} &= -\,(\mu + \gamma)\,V - \rho V - \lambda\,(1 - \mathrm{AVE}_S) + \sigma S \\
\frac{dI_2}{dt} &= -\,(\mu + \nu\,(1 - \mathrm{AVE}_I))\,I_2 - \alpha I_2 + \lambda\,(1 - \mathrm{AVE}_S)\,V\ .
\end{aligned}
$$

In compact matrix notation the dynamics of the model can be written as $\frac{d\mathbf{x}}{dt} = \mathbf{A}\mathbf{x}$,where $\mathbf{x} = (S, I_1, R, V, I_2)^T$ is the population state vector. The matrix $\mathbf{A}$ can be decomposed in a matrix $\mathbf{F}$ describing the birth of individuals and a transfer matrix $\mathbf{T}$: $\mathbf{A} = \mathbf{F} + \mathbf{T}$ [1]. Notice that the birth rate is denoted by b, and that all individuals are born in the susceptible class. The transfer matrix is given by:

$$\mathbf{T} = \begin{pmatrix} -\mu-\lambda-\sigma & 0 & 0 & \rho & 0 \\ \lambda & -\mu-\nu-\alpha & 0 & 0 & 0 \\ 0 & \alpha & -\mu & 0 & \alpha \\ \sigma & 0 & 0 & -\mu-\gamma-\rho-\lambda\,(1-\mathrm{AVE}_S) & 0 \\ 0 & 0 & 0 & \lambda\,(1-\mathrm{AVE}_S) & -\alpha-\nu\,(1-\mathrm{AVE}_I) \end{pmatrix} . \tag{A1}$$

Prevention

In a stationary population the reproduction number determines whether the pathogen can invade a population where it is not yet present. It is well known that the reproduction number is given by the largest eigenvalue of the (nonnegative) matrix **R**, the elements of which give the number of type 1/type 2 infections caused by a single type 1/type 2 infection over the infectious period of a type 1/type 2 infection [2]. **R** is given by

$$\mathbf{R} = \begin{pmatrix} \frac{\beta}{\mu+\nu+\alpha}\frac{\mu+\gamma+\rho}{\mu+\gamma+\rho+\sigma} & \frac{\beta(1-\mathrm{AVE}_I)}{\mu+\nu(1-\mathrm{AVE}_I)+\alpha}\frac{\mu+\gamma+\rho}{\mu+\gamma+\rho+\sigma} \\ \frac{\beta(1-\mathrm{AVE}_S)}{\mu+\nu+\alpha}\frac{\sigma}{\mu+\gamma+\rho+\sigma} & \frac{\beta(1-\mathrm{AVE}_S)(1-\mathrm{AVE}_I)}{\mu+\nu(1-\mathrm{AVE}_I)+\alpha}\frac{\sigma}{\mu+\gamma+\rho+\sigma} \end{pmatrix} , \tag{A2}$$

where β and $\beta\,(1-\mathrm{AVE}_I)$ denote the infection rates by type 1 and type 2 infected individuals in a population consisting of susceptibles only, and $\frac{\mu+\gamma+\rho}{\mu+\gamma+\rho+\sigma}$ and $\frac{\sigma}{\mu+\gamma+\rho+\sigma}$ denote the fraction of individuals in class S and class V in the pathogen's absence. A straightforward calculation shows that the reproduction number is given by

$$R_0 = \beta\left(\frac{1}{\mu+\nu+\alpha}\frac{\mu+\gamma+\rho}{\mu+\gamma+\rho+\sigma}+\frac{(1-\mathrm{AVE}_S)\,(1-\mathrm{AVE}_I)}{\mu+\nu\,(1-\mathrm{AVE}_I)+\alpha}\frac{\sigma}{\mu+\gamma+\rho+\sigma}\right) . \tag{A3}$$

In the case that the rate of antiviral mortality is small in comparison with either the background mortality μ or the recovery rate α, the infectious periods of type 1 and type 2 infection are almost equal, and the above equation can be approximated by

$$R_0 \approx \frac{\beta}{\mu+\alpha}\,\frac{\mu+\gamma+\rho+\sigma\,(1-\mathrm{AVE}_S)\,(1-\mathrm{AVE}_I)}{\mu+\gamma+\rho+\sigma} . \tag{A4}$$

In the case of a perfect antiviral drug ($\mathrm{AVE}_S = 1$) (A3) simplifies to

$$R_0 = \frac{\beta}{\mu+\nu+\alpha}\,\frac{\mu+\gamma+\rho}{\mu+\gamma+\rho+\sigma} , \tag{A5}$$

i.e. the reproduction number is given by the product of the reproduction number in a fully susceptible population ($\frac{\beta}{\mu+\nu+\alpha}$) times the fraction of individuals in the susceptible class in the absence of the pathogen ($\frac{\mu+\gamma+\rho}{\mu+\gamma+\rho+\sigma}$).

Early control
Simulations of the pathogen dynamics in a finite population are based on a stochastic counterpart of the deterministic model [3]. Briefly, the population is characterized by a vector describing the number of individuals in the various classes. Given a population composition at time t an inter-event time is drawn from an exponential distribution with parameter given by the sum of the rates of all possible events. Hence, the parameter of this distribution is given by

$$\begin{aligned} &\mu N + \gamma V + \nu \left(I_1 + \left(1 - \mathrm{AVE}_I\right) I_2 \right) + \sigma S + \rho V \\ &+ \beta \left(S + \left(1 - \mathrm{AVE}_S\right) V \right) \left(I_1 + \left(1 - \mathrm{AVE}_I\right) I_2 \right) + \alpha \left(I_1 + I_2 \right) \ . \end{aligned}$$

The mean of the inter-event time is given by the reciprocal of the above number. Subsequently, a specific event is chosen with probability given by the relative magnitude of the rate of the event. This procedure is repeated until there are no infected individuals anymore. In small populations, this approach is the method of choice.
Here, however, because of the large populations that we would like to handle the method becomes impractical, and we make use of a discrete time-step approximation. In the approximation, the number of transitions from S to V, $C_{S \to V}$, is given by

$$C_{S \to V} \sim Bin\left(S, 1 - e^{-\sigma \Delta t}\right) \ ,$$

i.e. the number of transitions from S to V in a fixed time interval Δt is binomially distributed with parameter $1 - \exp\left(-\sigma \Delta t\right)$ (the probability of transition of a single individual) and binomial totals S. Other transitions are handled in a similar manner.
In our simulations we assumed that $\sigma = 365$ (yr) once antiviral control is started, implying that a susceptible individual is on antiviral drugs after one day on average. In all simulations we used a time-step of one hour. Doubling the time-step gave identical results, indicating that the time-step was sufficiently small (results not shown).

The critical force of infection

For a fixed force of infection λ, the transfer of individuals over the classes is described by linear ordinary differential equations:

$$\frac{d\mathbf{p}}{dt} = \mathbf{T}\ \mathbf{p}\ . \tag{A6}$$

The solution of (A6) is given by $\mathbf{p}\,(t) = \sum c_i\ \mathbf{v}_i\ e^{\lambda_i t}$, where λ_i are the eigenvalues of $\mathbf{T}$ (which are all distinct), $\mathbf{v}_i$ are corresponding (right) eigenvectors, and c_i are constants which are determined by the initial conditions. Hence, with appropriate initial conditions $P\,(t) = \sum\limits_i p_i\,(t)$ gives the probability that an individual is still alive at time t. If the probability to remain alive is larger when initially in the susceptible rather than in the protected class it is better not to take antiviral drugs, while taking antiviral drugs is the rational strategy if the probability to remain alive is larger when initially in the protected (rather than the susceptible) class. Figure 3 is based on this approach.

To obtain some analytical insight in the optimal antiviral control strategies one may argue as follows. If the horizon t is small, the probability that an individual is alive can be approximated by a Taylor series of $P\,(t)$ in t around $t = 0$. Hence, the probability that an individual is alive given that it was initially in S or V is given by

$$P_S\,(t) = 1 - \mu t + \frac{1}{2}\left(\mu^2 + \nu\lambda\right)^2 t^2 + h.o.t.$$

and

$$P_V\,(t) = 1 - (\mu + \gamma)\,t + \frac{1}{2}\,(\mu + \gamma)^2\,t^2 + h.o.t.\ ,$$

respectively. Dropping higher order terms, equating $P_S\,(t)$ and $P_V\,(t)$ and solving for $\lambda = \lambda_c$ shows that

$$\lambda_c \approx \frac{2\gamma}{\nu}\left(\frac{1}{t} - \frac{1}{2}\gamma - \mu\right)\ . \tag{A7}$$

In addition to showing the analysis based on the exact solution of (A6), Figure 3 in the main text also shows the approximation (A7). It appears that for the default parameters the approximation (A7) works well if the horizon t is one week or less. If t is larger than one week, the approximation (A7) may substantially underestimate the λ_c, and higher order terms are needed to reliably approximate the critical force of infection.

At equilibrium, insertion of the force of infection ($\lambda = \frac{\mu(\beta-\alpha-\mu-\nu)}{\alpha+\mu}$) in (A7)

yields an expression for the critical horizon $t = t_c$ in terms of the model parameters:

$$t_c \approx \frac{2\gamma\left(\alpha+\mu\right)}{\mu\left(\beta-\alpha-\mu\right)\nu+\gamma\left(\alpha+\mu\right)\left(\gamma+2\mu\right)-\mu\nu^2}\ . \tag{A8}$$

If $t < t_c$ for a given set of parameters, it is better not to take antiviral drugs, while the reverse is true if $t > t_c$.
As an alternative approach, one could focus on maximization of life expectancy. A straightforward calculation shows that in this case the critical force of infection is given by

$$\lambda_c = \frac{\gamma\mu\left(\mu+\nu+\alpha\right)}{\nu\mu-\gamma\left(\mu+\alpha\right)}\ . \tag{A9}$$

Intuitively, (A9) can be understood as follows. The probability that an individual will die by infection is given by the product of the probability of infection ($\frac{\lambda}{\lambda+\mu}$) times the probability of death if infected ($\frac{\nu}{\nu+\mu+\alpha}$). The probability of death by antiviral treatment, on the other hand, is given by $\frac{\gamma}{\gamma+\mu}$. Hence, the probability of death by infection equals the probability of death by antiviral treatment if $\frac{\lambda_c}{\lambda_c+\mu}\frac{\nu}{\nu+\mu+\alpha} = \frac{\gamma}{\gamma+\mu}$. Solving this equation yields (A9).

Late control
In the long run the distribution of the individuals in the various classes ($\mathbf{w} = \left(w_S, w_{I_1}, w_R, w_V, w_{I_2}\right)^T$) is determined by a right dominant eigenvector of $\mathbf{A}$ or $\mathbf{T}$. To simplify the equations we will assume that antiviral drugs provide complete protection against infection ($\text{AVE}_S = 1$) and omit the class of type 2 infecteds. A straightforward calculation shows that with this assumption

$$\mathbf{w} \propto \begin{pmatrix} 1 \\ \frac{\lambda}{z+\mu+\nu+\alpha} \\ \frac{\alpha\ \lambda}{(z+\mu)(z+\mu+\nu+\alpha)} \\ \frac{\sigma}{z+\mu+\gamma+\rho} \end{pmatrix}\ , \tag{A10}$$

where z is the dominant eigenvalue of $\mathbf{A}$. The relative frequencies in the various classes are given by the normalized dominant right eigenvector $\mathbf{u} = \mathbf{w}/\left|\mathbf{w}\right| = \left(u_S, u_{I_1}, u_R, u_V\right)^T$.
Now we incorporate population regulation into the model. To this end we assume that the birth parameter b is not a constant but flexibly adjusted to

ensure that total population size remains constant. The differential equation that describes the evolution of total population size is given by

$$\frac{dN}{dt} = (b - \mu)\, N - \gamma V - \nu I_1 \ .$$

Notice that if population size is assumed to be constant ($z = 0$), the birth rate cannot be constant but should satisfy

$$b = \mu + \gamma\, \frac{V}{N} + \nu\, \frac{I_1}{N} \ .$$

Hence, in a population of constant size the birth rate equals to the natural death rate (μ) plus the antiviral prophylaxis and infection-induced mortality ($\gamma \frac{V}{N}$ and $\nu \frac{I_1}{N}$). Insertion of $u_V = \frac{V}{N}$ and $u_{I_1} = \frac{I_1}{N}$ (using (A10) with $z = 0$) in the above equation yields an expression for b in terms of the force of infection λ.

To incorporate the infection feedback loop we let the force of infection depend on the prevalence of infection as follows:

$$\lambda = \beta\, u_{I_1} \ , \tag{A11}$$

where β is the transmission rate parameter. Insertion of (A11) into (A10) and solving the resulting equations for u_{I_1} allows one to obtain an expression for the prevalence of infection in terms of the parameters of the model:

$$u_{I_1} = \frac{\mu}{\mu + \rho} \left(1 - \frac{\mu + \alpha + \rho}{\beta} \left(1 + \frac{\nu}{\mu + \gamma + \sigma} \right) \right) \ , \tag{A12}$$

unless $R < 1$ in which case $u_{I_1} = 0$. Explicit expressions for u_S, u_R, and u_V are calculated in the same manner.

In the more general case where individuals in the protected class can still be infected ($\mathrm{AVE}_S < 1$), (A11) is replaced by

$$\lambda = \beta \left[u_{I_1} + (1 - \mathrm{AVE}_I)\, u_{I_2} \right] \ . \tag{A13}$$

A procedure similar to the one described above yields a cubic equation for the force of infection in terms of the model parameters. Solving the equation yields explicit, though complicated, expressions for the prevalence of type 1 and type 2 infections at equilibrium.

We would like to remark that, from a mathematical point of view, the above derivation and analysis of the model are not fully rigorous and complete but based on a number of handwaving arguments. A more rigorous treatment

of epidemic ordinary differential equations models is given in, e.g., [4].
In case of a perfect antiviral drug the mortality incidence at equilibrium D caused by both infection and antiviral prophylaxis is given by

$$D = \nu u_{I_1} + \gamma u_V \ , \tag{A14}$$

where u_{I_1} and u_V are calculated using the procedure described above. Insertion of u_{I_1} and u_V in (A14) yields

$$D = \frac{\mu\left(\mu+\nu+\alpha\right)\left(\sigma\left(\mu+\gamma\right)+\left(\mu+\lambda\right)\left(\mu+\gamma+\rho\right)\right)}{\sigma\mu\left(\mu+\nu+\alpha\right)+\left(\left(\mu+\lambda\right)\left(\mu+\alpha\right)+\mu\nu\right)\left(\mu+\gamma+\rho\right)} - \mu \ . \tag{A15}$$

If we further insert the explicit equation for the force of infection (using (A11) and (A12)) in the above equation, we get an expression for D in terms of the basic parameters of the model. A straightforward but tedious calculation shows that D is given by

$$D = \frac{\mu\nu\left(\beta-\left(\mu+\nu+\alpha\right)\right)\left(\mu+\gamma+\rho\right)+\sigma\left(\mu+\nu+\alpha\right)\left(\gamma\left(\mu+\alpha\right)-\mu\nu\right)}{\beta\left(\mu+\alpha\right)\left(\mu+\gamma+\rho\right)} \ . \tag{A16}$$

Since all factors in (A16) are positive except for $\gamma\left(\mu+\alpha\right)-\mu\nu$ in the numerator, it follows that D increases with increasing σ if $\gamma\left(\mu+\alpha\right)-\mu\nu > 0$. Otherwise, D decreases with increasing σ. This proves equation (2) in the main text.

The individual versus population perspective
Armed with (A16) we are in the position to determine which antiviral control rate minimizes excess mortality. In fact, inspection of (A16) shows that if $\gamma < \frac{\mu}{\mu+\alpha}\nu$ excess mortality is minimal if the pathogen is just unable to invade. Hence, the optimal antiviral rate is given by the solution of the equation $R = 1$ (A5), which yields

$$\sigma^*_{pop} = \left(\mu+\gamma+\rho\right)\left(\frac{\beta}{\mu+\nu+\alpha} - 1\right) \ , \tag{A17}$$

unless $\gamma > \frac{\mu}{\mu+\alpha}\nu$ in which case $\sigma^*_{pop} = 0$.
To determine the optimal individual control rate we focus on a (vanishingly) small number of individuals that use a control rate σ_y in a population where the (vast) majority of individuals use a control rate σ_x. Using (A15) we can calculate excess mortality of the subpopulation of σ_y-individuals in a

population of σ_x individuals. Excess mortality is given by (A15), but with σ replaced with σ_y, and λ replaced with $\lambda[\sigma_x]$:

$$D(\sigma_y, \sigma_x) = \frac{\mu(\mu+\nu+\alpha)(\sigma_y(\mu+\gamma)+(\mu+\lambda[\sigma_x])(\mu+\gamma+\rho))}{\sigma_y\mu(\mu+\nu+\alpha)+((\mu+\lambda[\sigma_x])(\mu+\alpha)+\mu\nu)(\mu+\gamma+\rho)} - \mu \ .$$

An expression for the force of infection $\lambda[\sigma_x]$ in terms of the basic parameters is obtained by insertion of (A10) and (A11) in the above equation:

$$D(\sigma_y, \sigma_x) = \frac{\mu\nu(\beta-(\mu+\nu+\alpha))(\mu+\gamma+\rho)+(\mu+\nu+\alpha)(\sigma_y\gamma(\mu+\alpha)-\sigma_x\mu\nu)}{(\mu+\alpha)(\beta(\mu+\gamma+\rho)+(\sigma_y-\sigma_x)(\mu+\nu+\alpha))} \ . \tag{A18}$$

Similar calculations allow one to determine excess mortality in case of an imperfect antiviral drug (results not shown).

In the main text (Figures 5 and 6) we have plotted the local minima of $D(\sigma_y, \sigma_x)$ in σ_y at $\sigma_y = \sigma_x$. Extrema of (A18) can be found by solving $\left.\frac{\partial D(\sigma_y,\sigma_x)}{\partial \sigma_y}\right|_{\sigma_y=\sigma_x=\sigma^*_{ind}} = 0$ [5]. The calculations yield

$$\sigma^*_{ind} = \frac{(\mu+\gamma+\rho)\left(\nu\mu(\beta-\mu-\alpha)-\beta\gamma(\mu+\alpha)-\nu^2\mu\right)}{(\mu+\nu+\alpha)(\nu\mu-\gamma(\mu+\alpha))} \ . \tag{A19}$$

In generic cases the sign of $\left.\frac{\partial^2 D(\sigma_y,\sigma^*_{ind})}{\partial \sigma_y^2}\right|_{\sigma_y=\sigma^*_{ind}}$ determines whether σ^*_{ind} minimizes or maximizes (A18)[5]. Notice that σ^*_{ind} corresponds to a Nash strategy and even an Evolutionarily Stable Strategy if it minimizes (A18). Numerical inspections indicate that σ^*_{ind} minimizes (A18) if equation (2) in the main text holds. If equation (2) does not hold it is best not to take antiviral drugs ($\sigma^*_{ind} = 0$).

References

1. Caswell H (2001) Matrix Population Models. Construction, Analysis, and Interpretation. Sinauer, Sunderland

2. Diekmann O, JAP Heesterbeek (2000) Mathematical Epidemiology of Infectious Diseases. Wiley, Chichester

3. Gillespie DT (1977) Exact Stochastic Simulation of Coupled Chemical Reactions. The Journal of Physical Chemistry 81, 2340-2361

4. Hadeler KP (1992) Periodic solutions of homogeneous equations. Journal of Differential Equations 95, 183-202

5. Otto SP, T Day (2007) A Biologist's Guide to Mathematical Modeling. Princeton University Press, Princeton
